# Improving Biodegradation of Clofibric Acid by *Trametes pubescens* through the Design of Experimental Tools

**DOI:** 10.3390/microorganisms8081243

**Published:** 2020-08-15

**Authors:** Claudia Veronica Ungureanu, Lidia Favier, Gabriela Elena Bahrim

**Affiliations:** 1 Cross-Border Faculty, Dunărea de Jos University, 800008 Galati, Romania; 2 Ecole Nationale Supérieure de Chimie de Rennes, CNRS, UMR 6226, Université Européenne de Bretagne, CEDEX 7, 35708 Rennes, France; lidia.favier@ensc-rennes.fr; 3 Faculty of Food Science and Engineering, Dunărea de Jos University, 800008 Galati, Romania

**Keywords:** clofibric acid, *Trametes pubescens*, biodegradation, Plackett-Burman design, response surface methodology, pharmaceutical active compound (PhAC)

## Abstract

Clofibric acid (CLF) is the main pharmacologically active metabolite in composition of the pharmaceutical products used for controlling blood lipid content. This xenobiotic compound is highly persistent in the aquatic environment and passes unchanged or poorly transformed in wastewater treatment plants. A white-rot fungal strain of *Trametes pubescens* was previously selected, for its ability for clofibric acid biodegradation (up to 30%) during cultivation in submerged system under aerobic conditions at an initial CLF concentration of 15 mg L^−1^. Plackett-Burman design (PBD) and response surface methodology (RSM) were used for experimental planning, mathematical modelling and statistical analysis of data of the biotechnological process of CLF biotransformation by *Trametes pubescens* fungal strain. After optimization, the capacity of the selected *Trametes pubescens* strain to degrade CLF was increased by cultivation in a liquid medium containing 3 g·L^−1^ yeast extract, 15 g·L^−1^ peptone, 5 g·L^−1^ glucose and mineral salts, inoculated at 2% (*v/v*) vegetative inoculum and cultivated at pH 5.5, during 14 days at 25 °C and 135 rpm. In these optimized biotechnological conditions, the CLF biotransformation yield was 60%.

## 1. Introduction

Organic contaminants in the aquatic environment have long been issues of environmental concern. While some of the first pollutants to be observed included pesticides and herbicides, recently pharmaceuticals have received much attention based on their toxicity against biological systems, even in concentrations of ng·L^−1^ [1]. As aquatic pollutants, these drugs are xenobiotic compounds and have moderate and high recalcitrance in correlation with their chemical structure and toxicity.

Clofibric acid (CLF) is one of the most often reported drug metabolite detected in the aquatic environment during the monitoring programs of pharmaceutical contaminants. Several studies reported the presence of this molecule in wastewater treatment plants (WWTP). CLF is also used as a herbicide (Mecoprop or MCPP and MCPA), so large amounts are to be expected coming not only from the medical industry, but also from agricultural fields (soils, water channels, phreatic filtrations, etc.) [2,3].

Conventional physicochemical process, such as advanced oxidation process, activated carbon adsorption and UV/H_2_O_2_ induced photolytic degradation, give better results of CLF degradation (>90%), but the inherent drawbacks due to the tendency of the formation of secondary toxic by-products and the cost related to these kind of advanced technologies are significant and they pose maintenance problems which make them economically unfeasible for many municipalities [4,5,6,7]. Bioremediation techniques, based on augmentation strategy, have received the most attention, because it is friendly to environment and implies relatively low cost [8]. The potential of microorganisms to catabolize and metabolize xenobiotic compounds has been recognized as a potentially effective means of toxic and hazardous waste disposal [9].

The white-rot fungi (WRF) are a cosmopolitan group of microorganisms with a high capability to degrade a wide range of xenobiotic and recalcitrant pollutants due to their ability to biosynthesis of extracellular manganese and lignin peroxidase, laccase and versatile peroxidases. The manganese peroxidase and laccase enzymes are metal enzymes which are contain Cu^+2^ and Mn^2+^ in their structures. In this context the MnSO_4_·H_2_O and CuSO_4_·5H_2_O have an essential role as precursors in the fermentation medium [10,11]. Their ability being of great interest for the development of environmentally friendly biotechnological processes to be applied in organic persistent pollutants elimination by bioremediation.

Only few studies showed the ability of white-rot fungi like as *Trametes versicolor*, *Irpex lacteus, Ganoderma lucidum* and *Phanerochaete chrysosporium*, to degrade pharmaceutical residues, like carbamazepine, ibuprofen and clofibric acid. Thus, for a *Trametes versicolor* selected strain was demonstrated the capacity to biodegradation of CLF, up to 97%, after seven days of aerobic cultivation in liquid medium containing 10 mg·L^−1^ of PhAC [12]. Previously, one *Trametes pubescens* strain was selected, by Ungureanu et al. [13], for its ability to degrade CLF (up to 30%), during cultivation in submerged system under aerobic conditions at an initial concentration of 15 mg·L^−1^.

Currently, the ability of microorganisms to degrade toxic molecules is strongly affected by a many experimental factors such as nutritional requirements, cells energetic status and physico-chemical culture conditions. However, there is no knowledge about nutritional and environmental requirements for CLF degradation by *Trametes pubescens*. Therefore, it is necessary to design an appropriate process for improving the CLF degradation efficiency by *Trametes pubescens*.

Also, the investigation of the effects of certain of these factors should indicate the optimal experimental conditions that play a role in the bioremediation process.

Applied of the design of experiments (DOE) tools are versatile techniques for investigation of multiple process variables because it makes the process easily optimized with fewer experimental trials [14]. The Plackett-Burman design (PBD) provides a fast and effective way to screening a large number of variables and identifying the most important ones [15]. Central composite design (CCD) and response surface methodology (RSM), which includes factorial design and regression analyses, helps in planning of experiments and evaluating the important factors, building models to reveals the interactions between the independent variables and responses [16].

The main aim of this work was to increase the biodegradation efficiency of CLF using a selected strain of *Trametes pubescens*, by optimizing the cultivation conditions (nutritional and environmental factors) using mathematical modelling and statistical approaches associated with PBD and RSM guided design of experiments.

## 2. Materials and Methods

### 2.1. Chemicals and Fungal Strain

The white-rot fungal strain *Trametes pubescens*, was provided from the Cultures Collection of the Faculty of Biology of the *Alexandru Ioan Cuza* University of Iasi, Romania. This strain is now in the gestion of the Collection of Microorganisms of *Dunărea de Jos* University of Galați Romania, with acronym MIUG. The stock culture is preserved by cryoconservation in 40% glycerol at −80 °C. For reactivation, the strain was cultivated on 2% malt extract agar slant (pH = 4.5) at 25 °C for 5 days [17].

Analytical grade chemical reagents, CLF, methanol (HPLC grade), ingredients and culture media were purchased from Sigma-Aldrich (St. Louis, MO, USA).

### 2.2. Bioremediation Experiments

The biodegradation experiments were performed by cultivation in basal liquid medium (MM) contains (g·L^−1^): MnSO_4_·H_2_O, 0.50; CuSO_4_·5H_2_O, 0.50, FeSO_4_·7H_2_O, 0.01, MgSO_4_·7H_2_O, 0.50; ZnSO_4_·7 H_2_O, 0.005; pH = 5.5, supplemented with organic nitrogen and carbon sources (yeast extract, peptone and glucose) [18]. For optimization studies, the studied independent variables such as: initial concentrations of clofibric acid, nitrogen sources and glucose, MnSO_4_·H_2_O, CuSO_4_·5H_2_O, inoculum level and incubation time, were varied according to the experimental design (Table 1).

A mycelial suspension of *Trametes pubescens* was obtained by inoculation of three agar plugs (6 mm in diameter), collected from the growing zone of fungi on plates of malt extract agar, to a 500 mL Erlenmeyer flask containing 150 mL of MEG broth. The cultivation took places on a rotary shaker SI-300R Incubator Shaker (Jeio Tech, Korea) at 135 rpm and temperature of 25 °C. After 4–5 days, the dense mycelial mass was aseptically blended with a homogenizer (Waring blender, Germany), to obtain blended mycelial inoculum. Pellets of *Trametes pubescens* were produced by inoculating 1 mL of the mycelial suspension in 150 mL of MM liquid medium, which was shaken (135 rpm) at 25 °C for 5 days and the mycelial pellets obtained were washed with deionized water. In the performed experiments, the concentration of the mycelial pellets varied between 0.5% and 2%.

Samples were aseptically taken at regular intervals and investigated for biomass yield production (expressed as dry weight) and residual CLF concentration.

Also, biotic and abiotic controls were also performed to monitoring the abiotic degradation of CLF and evaluation the possible adsorption of this on the cell biomass [13].

### 2.3. Biomass Dry Weight Assay

The biomass concentration was determined by dry weight of fungal mycelium. The culture medium was vacuum filtered through 0.45 μm glass microfiber filters (Whatman GF/C, Maidstone, England). The filters containing mycelial mass were placed in glass dishes and dried at 100 °C (Sanyo drying Oven, Japan) to constant weight.

### 2.4. HPLC Analysis

The crude culture supernatants, after biomass separation by centrifugation at 10,000 rpm for 10 min was filtered through a membrane filter of polyester (PALL Life Science, New York, NY, USA, 0.2 μm) and then transferred to HPLC vials.

The residual pollutant concentration was determined by high performance liquid chromatography (HPLC) Agilent 1200 Series (Santa Clara, CA, SUA) equipped with a photodiode array (PDA) detector operating at a wavelength of 230 nm. Chromatographic separation was achieved on a C18 column (150 mm × 4.6 mm, particle 5 µm) at 40 °C.

The mobile phase consisted of a mixture of methanol: ultrapure water (70:30, *v/v*) and 0.1% acetic acid at a flow rate of 1.0 mL min^−1^. The retention time was 2.4 min and the instrumental quantification limit (LOQ) for CLF was <0.2 mg L^−1^.

CLF concentration (expressed as mg L^−1^) was calculated using an external calibration curve, which was carried out using standard solutions with a known analyte concentration (in the range of 10–30 mg L^−1^).

Biotransformation yield of CLF was calculated as the percentage of the quantity of the CLF bioconversion after 14 days of incubation (mg L^−1^) in ratio with the initial CLF concentration (mg L^−1^).

### 2.5. Design of Experiments Methodology

#### 2.5.1. Screening of Important Biotechnological Parameters by Plackett-Burman Design

The PBD was used to screen and evaluate the important medium components with significant influence on the CLF biotransformation, under aerobic cultivation conditions in submerged system, by *Trametes pubescens* strain.

The first-order polynomial model was used (Equation (1)):

(1) $$Y=\beta_{0}+\Sigma \beta_{i}\chi_{i}$$

where: *Y* is the predicted response (CLF biotransformation yield), *β*_0_, *β_i_* are constant coefficients and *χ_i_* is the coded independent variables.

Based on Plackett-Burman design (PBD), each independent variables were evaluated at low (−1) and high (+1) levels (Table 1).

All tests were performed in triplicate and the averages of the results were taken as response values.

The factors significant at 95% level (*p* < 0.05) were considered to have significant effect on CLF biotransformation and the variable with positive influence was used for further optimization by RSM.

#### 2.5.2. Central Composite Design (CCD) and Response Surface Methodology (RSM)

The information about the significant effects and the interactions between the selected parameters with positive influence on CLF biodegradation were analyzed and optimized by CCD and RSM techniques. The effect of yeast extract concentration (B), peptone concentration (C), the inoculum concentration (F) and the incubation time (H) on the percentage of CLF degradation was evaluated by mathematical optimization and statistical analysis, by using five experimental levels: −α, −1, 0, +1, +α. The experimental levels were selected by varying the parameters above and below the central point (Table 2).

The mathematical relationship between the independent variables and the response was calculated by the following quadratic (second degree) polynomial equation (Equation (2)):

*Y* = *β*_0_ + *β*_1_B + *β*_2_C + *β*_3_F + *β*_4_H + *β*_12_BC + *β*_13_BF + *β*_14_BH + *β*_23_CF + *β*_24_CH + *β*_34_FH + *β*_11_B^2^ + *β*_22_C^2^ + *β*_33_F^2^ + *β*_44_H^2^ (2)

where: *Y* is the response (CLF biotransformation yield); B, C, F, H are the code of the independent variables; *β*_1_, *β*_2_, *β*_3_, *β*_4_, are linear regression coefficients; *β*_11_, *β*_22_, *β*_33_, *β*_44_, are quadratic regression coefficients; *β*_12_, *β*_13_, *β*_14_, *β*_23_, *β*_24_, *β*_34_, are interactive regression coefficient estimates while *β*_0_ have a role of scaling constant.

### 2.6. Statistical Analysis

Design Expert software (version 9.0.4.1, State-Ease, Inc., Minneapolis, MN, USA) was used for experimental design and regression analysis of the experimental data.

Analysis of variance (ANOVA) was employed to estimate the statistical parameters. This analysis included Fischer’s F-test, its associated probability p (F), the correlation coefficient (R^2^), determination coefficient (R^2^) which measures the goodness of fit of regression model.

## 3. Results

### 3.1. Selection of Significant Biotechnological Parameters on the CLF Biodegradation by Using PBD

The influence of the eight different independent variables on the CLF biotransformation in aerobic submerged cultivation system of a selected strain of *Trametes pubescens* was studied by using PBD methodology. The design matrix selected for the screening of independent variables for CLF biodegradation and the corresponding response in term of percentage of biotransformation yield are given in Table 3.

The data listed in Table 3 indicated a wide variation in CLF biodegradation yields in the range of 20–100%, in the 12 trials. After 14 days of aerobic submerged cultivation, the CLF from liquid medium was degraded up to 100%, started from an initial pollutant concentration of 10 mg·L^−1^, while the CLF degradation yield at an initial concentration of 15 mg·L^−1^ was much lower (20%).

**Figure 1:**
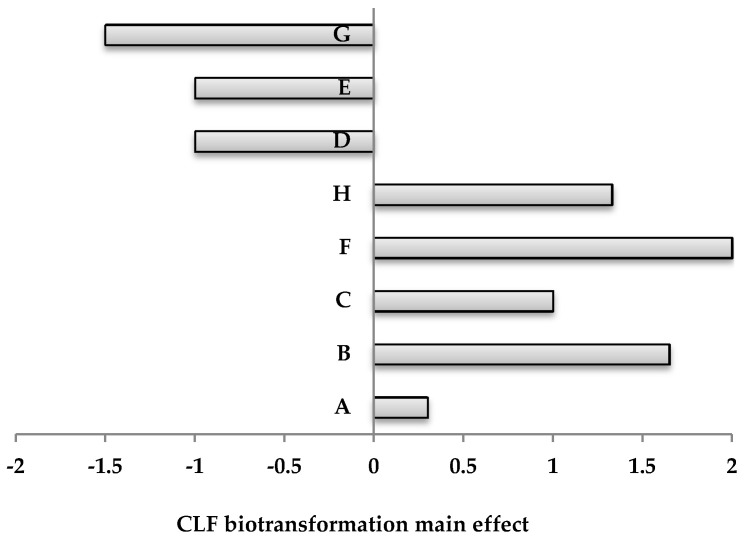
Pareto chart for the effect of independent variables on CLF biotransformation by *Trametes pubescens* based on PBD analysis.

A large contrast mean, either positive or negative, indicates that a factor has a large impact on CLF biotransformation; while a mean close to zero means that the factor has little or no effect (Figure 1).

Thus, based on the statistical analysis was established that the factors having a positive influence on biotransformation process are: the concentration of yeast extract (B), of peptone (C), of inoculum (F) and incubation time (H) From the results obtained in this experiment, it was observed that 5 g·L^−1^ glucose favored the CLF removal. Other parameters as CuSO_4_·5H_2_O concentration (D) and MnSO_4_·H_2_O concentration (E) and initial CLF concentration (G) possess a negative influence at studied concentration and analyzed levels of variation (Figure 1).

### 3.2. Model Fitting and Statistical Analysis

The variables showing confidence level greater than 95% in PBD were selected for further optimization using CCD method. The levels of the factors chosen were set based on the previous PBD analysis. Each of the independent variable was studied at five coded levels (−α, −1, 0, +1, +α) with 30 experiments and all variables were taken at a central coded value of zero. To determine their optimum levels for maximum biodegradation yield demonstrated markedly varied results, ranging from 0–80%. The lowest biodegradation yield was observed when the culture medium was not inoculated. High biotransformation level (60%) was obtained in a cultivation medium containing 3 g·L^−1^ yeast extract, 15 g·L^−1^ peptone, 5 g·L^−1^ glucose, pH = 5.5, 2% inoculum concentration after 14 days of submerged cultivation at 25 °C and 135 rpm. The design matrix with the experimental results is shown in Table 4.

The statistical model was developed by applying multiple regressions analysis on the experimental data the following second-order polynomial equation was established to describe the CLF biodegradation (Equation (3)):

*Y* = 46.67 + 5.42 *B* + 2.50 *C* + 11.67 *F* + 14.17 *H* − 1.88 *BC* + 1.25 *BF* + 5.00 *BH* + 1.88 *CF* + 0.63 *CH* + 3.75 *FH* + 0.21 B^2^ − 3.96 *C*^2^ + 3.33 *F*^2^ + 3.33 *H*^2^ (3)

where: *Y* was the predicted CLF biotransformation yield (%), *B* the concentration of yeast extract (g·L^−1^), *C* the concentration of peptone (g·L^−1^), *F* the inoculum concentration (%, *v/v*) and *H* the incubation time.

The incubation time (*H*) has the highest regression coefficient (14.17), followed by the inoculation level (11.67), the concentration of yeast extract (5.42) and the concentration of peptone (2.50) and these independent variables are considered with influence on CLF biodegradation.

The adequacy of the model was checked by *F*-test and the analyses of variance (ANOVA) for the response surface quadratic model are presented in Table 5. The model *F* value of 15.36 implied that the model was significant and also showed that there was only 0.05% chance that a “model *F*-value” could occur due to noise. Furthermore, the lack of fit test is performed by comparing the variability of the current model residuals to the variability between observations at replicate settings of the factors. The lack of fit value of 2.26 implies that the lack of fit is not significant relative to the pure error (lack of fit *p* > 0.05).

The *p* values (Prob > F) less than 0.050 denoted the significance of the coefficients and it is also important in understanding the pattern of the mutual interactions between the variables. In addition, *p* values greater than 0.05 indicates that the model terms are insignificant. The results presented in Table 5 showed that two interaction terms coded *BC* and *CH* and one quadratic term coded *B*^2^ can be removed.

**Figure 2:**
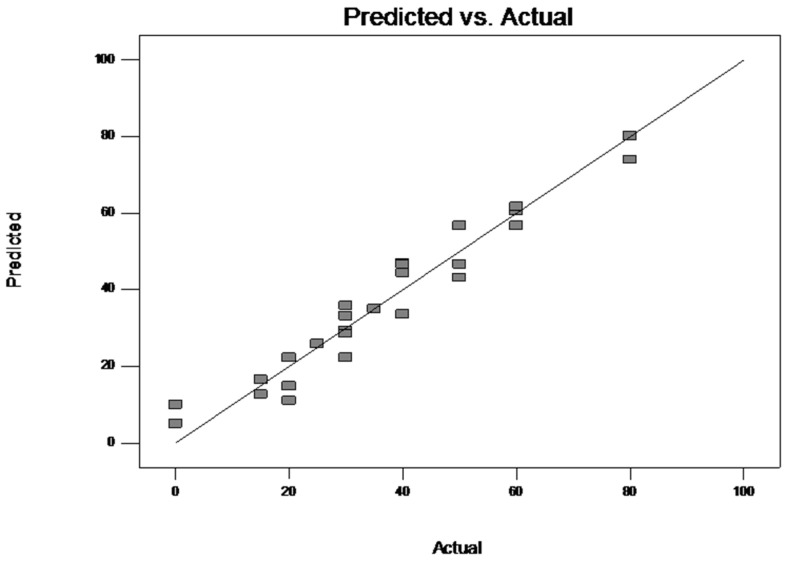
Parity plot of experimental vs. predicted values of CLF biodegradation by *Trametes pubescens*.

The goodness of fit of the model was checked by the determination coefficient *R*^2^ and the adjusted *R*^2^ (multiple correlation coefficient *R*). The *R*^2^ value indicated that 96.48% of the total variation was explained by the model. A regression model with *R*^2^ closed to 1.0 is considered as having a very high correlation. The value of the adjusted determination coefficient (Adj *R*^2^ = 90.40%) confirmed the significance of the model as well. In Figure 2 is presented, the correlation between the experimental and predicted values of percentage of CLF degradation, wherein, the points cluster around the diagonal line which indicates an optimal fit of the model, since the deviation between the experimental and predicted values was minimal.

The parameters with significant model terms were the linear terms of the concentration of yeast extract concentration (B), peptone concentration (C), inoculum concentration, (F) incubation time (H) and quadratic terms (C^2^), (F^2^), (H^2^) followed by the interactions effects terms (BF), (BH), (CF) and (FH). To investigate the interactions of the parameters required for optimum biodegradation yield the fitted response surface was generated by statistically significant above model by Design Expert program.

**Figure 3:**
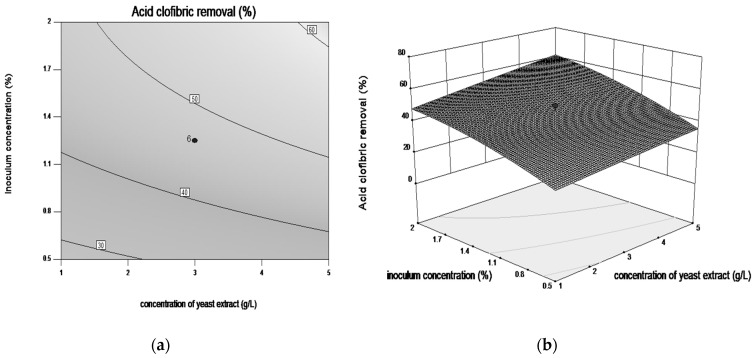
Contour plot (**a**) and three-dimensional surface plot (**b**) showing the effect between inoculum concentration and concentration of yeast extract on CLF biodegradation by *Trametes pubescens*.

**Figure 4:**
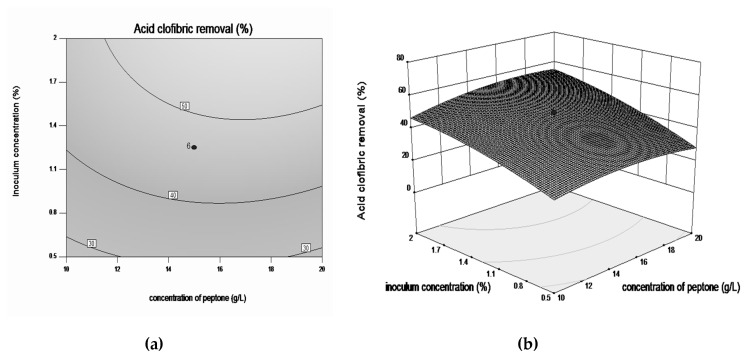
Contour plot (**a**) and three-dimensional surface plot (**b**) showing the effect between inoculum concentration and concentration of peptone on CLF biodegradation by *Trametes pubescens*.

**Figure 5:**
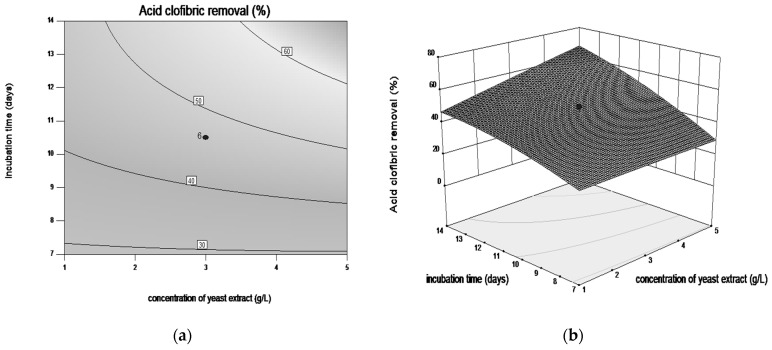
Contour plot (**a**) and three-dimensional surface plot (**b**) showing the effect between incubation time and concentration of yeast extract on CLF biodegradation by *Trametes pubescens*.

**Figure 6:**
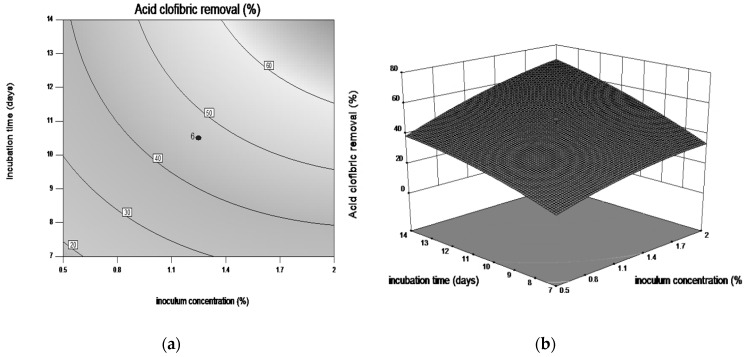
Contour plot (**a**) and three-dimensional surface plot (**b**) showing the effect between incubation time and inoculum concentration on CLF biodegradation by *Trametes pubescens*

Response surface plots and contour plots are shown in Figure 3, Figure 4, Figure 5 and Figure 6, which depict the interaction between two factors by keeping the other factors at their zero levels for biodegradation process.

Figure 3a,b and Figure 4a,b, illustrate the effects of interaction between concentration of nitrogen sources and the inoculum concentration. In these conditions the highest CLF degradation yield (up to 60%) was observed in the following ranges of the exanimated variables: 2% inoculum, 3 g·L^−1^ yeast extract and 15 g·L^−1^ peptone. The plots demonstrate that both nitrogen sources induce obtaining the best performance at optimum concentrations. It was also observed that excessive nutrients concentration does not have much effect on increasing the bioremediation yield.

The effect of interaction of incubation time and the concentration of yeast extract is illustrated in Figure 5a,b. The maximum bioremediation yield was achieved in medium with 3 g·L^−1^ yeast extract, after 14 days of submerged cultivation in aerobic conditions. Further, increase the concentration of this nitrogen source (>3 g·L^−1^), occurred a significant decrease of the bioremediation yield.

Figure 6a,b shows the response surface 3D of the effect of interaction between incubation time and inoculum concentration. The highest degradation yield (60%) was observed for 2% (*v/v*) inoculum concentration after 14 days of cultivation.

## 4. Discussion

The optimization of the *Trametes pubescens* cultivation medium, in order to increase the degradation yield of CLF, was performed by applying the central composite rotatable design (CCRD) of PBD and RSM statistical techniques. These tools have been successfully used in different biotechnological applications which target the process optimization [19,20].

Microorganisms acquire nutrients, ions and energy from their environments to support growth. Biodegradation of organic substrates provide microorganisms with energy and building materials that are used for growth of new cells, cell maintenance and co-metabolism of other less degradable substances [21].

In general, microorganisms grow mostly in media supplemented with additional substrates [22]. Hence, growth could be manipulated by addition of two or more nutrients simultaneously [23,24,25,26]. If a microbial population is grown on mixed substrates present in the medium, the microbes consume only one or both the substrates. Consequently, several utilization patterns can be observed. In mixed substrates, individual substrates can have synergistic, antagonistic or no effect on one another, resulting in a growth rate that is higher, lower or the same than if the substrates are present individually [27,28].

Biotic and abiotic tests (with biomass inactivated by autoclaving) were carried out in order to study the biosorption of the target compound and the biotransformation ability of selected strain was demonstrated. Moreover, controls were also done in order to monitor the abiotic degradation. The obtained results of biosorption tests indicated that this phenomenon is negligible (less than 3%) for all of the tested conditions. This value was considered for our calculations in order to determine the real biodegradation yield.

In a first instance, the significant biotechnological parameters that influence on the CLF biodegradation were selected using PBD technique. The statistical analysis evidenced that the MnSO_4_·H_2_O and CuSO_4_·5H_2_O must be used in small concentration in the fermentation medium to be effective inducers for enzymes implied in CLF biotransformation.

In addition, it can be noticed that the initial CLF concentration is the parameter which shows the most significant effect on biodegradation potential of the studied fungal strain.

It was reported that increasing the concentration of a pharmaceutical compound as residue pollutant may led to a toxic effect or decreasing the available oxygen and water activity of the medium upon physiologic function of the microorganisms, as well as, lowering the contact between the active biomass and nutrients [29]. Moore-Landeker [30] indicated that microorganisms differ between each other’s in tolerance of higher levels of target compound.

Moreover, it should be pointed out that the CLF concentrations considered in these tests are much higher than those detected in the influents of wastewater treatment plants (in the range of µg·L^−1^). However, it is also important to take into account that pharmaceuticals are not always detected at trace levels in polluted environments. Larsson et al. [31] reported that effluents from health care industry can contain high concentrations of pharmaceuticals. It is important to remark that the concentrations tested in this study (10 and 15 mg·L^−1^) are considerably higher than those reported in wastewater effluents (commonly in the µg·L^−1^ range). This aspect is important in selection of strains able to be tolerant at high concentrations of toxic, in order to be more effective in natural conditions in competition with wild microbiota. For this reason, in this study the initial pollutant concentration at the value considerate in the experimental design (15 mg·L^−1^) was chosen.

Among various nutritional requirements, carbon and nitrogen sources are generally regarded as important factors in co-metabolism of xenobiotic and recalcitrant compounds.

Naturally occurring carbon sources can have a significant impact on the ability of microbial communities to degrade pollutants. In this study, presence of the 5 g·L^−1^ glucose in cultivation medium composition it was efficiently for CLF biodegradation. Our study agrees whit the others reported in literature. Tran et al. [32] found that the presence of acetate (100 mg·L^−1^) as an additional carbon source increases the CLF removal. In their previous work, Ungureanu et al. [13] observed the degradation of CLF by ligninolytic fungal strains of *Trametes versicolar*, *Trametes pubescens*, *Irpex lacteus and Lenzites betulina* in the culture media supplemented with glucose. Likewise, *Streptomyces* spp. bacteria isolated from soils co-metabolized CLF and carbamazepine in the presence of glucose as additional source of carbon and energy [33,34,35]. Hemidouche et al. [36] showed that *Pseudomonas aeruginosa RZS9* is able to remove the CLF (up to 35%) in the presence of 2 g·L^−1^ of glucose.

In addition, the effects of glucose supply as well as light have been reported to play a significant role in fungal growth as well in diclofenac sodium removal efficiency [21].

Moreover, Wang and Loh [37] and Fakhruddin and Quilty [38] reported that high concentrations of glucose caused significant drop in pH and the inhibition of the assimilation of other substrates present in the culture medium.

The nitrogen requirement of five isolate South African indigenous fungal strains (*Trichoderma longibrachiatum, Trametes polyzona, Aspergillus niger, Mucor circinelloides* and *Rhizopus microspores*) showed that ammonium tartrate dibasic (AT) was the best nitrogen source, when compare to peptone as reported by Dutta and Das [39]. Also, Popa Ungureanu et al. [34] reported that nitrogen sources like yeast extract induced a rapid growth and accelerate the metabolism for *Streptomyces* spp. cultures implied in carbamazepine biodegradation. The nutrients requirement for optimum degradation depends on the microbial species employed and their metabolic and physiological behavior.

The inoculum size was reported in literature as an important factor for xenobiotic compounds degradation. This finding is consistent with many previous reports on the increase in the degradation percentage with increase in the inoculum size [40,41,42]. In our study, it was demonstrated that 2% vegetative fungal inoculum is effective for CLF biodegradation.

In addition, the pH of the medium remained almost constant during the biodegradation tests. This result indicates the absence of degradation metabolites usually formed during biodegradation processes which cause pH modification of the culture medium. Moreover, it confirms the data obtained on the removal of the target molecule.

White rot-fungi are a group of microorganisms with a high ability to degrade a wide range of organic pollutants such as synthetic dyes, polycyclic aromatic hydrocarbons, pharmaceuticals, personal care products and endocrine disrupting chemicals [43,44,45]. This ability is related to the production of oxidative enzymes (manganese and lignin peroxidase, laccase and versatile peroxidases) [46,47].

Previously, studies demonstrated that laccase was not involved in the degradation of CLF suggesting that this can be used only as an indicator for fungus activity [35,48]. In addition, it was demonstrated that the high concentration of CLF (15 mg·L^−1^) has a negative influence on production of enzymes implied in biodegradation [13]. In addition, other ligninolytic enzymes have been assumed to be involved in pharmaceuticals compound degradation by white-rot fungi [49].

Another enzymatic mechanism involved in degradation of xenobiotic compound by white-rot fungi is the CYT P 450 system (cytochrome P 450). Marco-Urrea et al. [12] reported that intracellular enzymatic system plays a major role in the first step of CLF oxidation by *Trametes versicolor* strains. Other studies showed that cytochrome P450 is also involved in the degradation of biphenyl, phenol, benzo(a)pyrene, aniline and pyridine [50].

According to literature data, the molecular structure of CLF is involved in its recalcitrant behavior and its resistance to biodegradation is in fact due to the steric hindrance from a single extra methyl group [51]. It is acknowledged that chlorinated substitutions or structural characteristics such as not only ring substitutions but also the nature of aliphatic side chain of compounds influence their persistence in the environment, affecting their susceptibility to biological degradation.

Thus, study of the influence of experimental parameters should indicate the optimal experimental conditions that play a key role in the bioremediation process and lead to a maximum removal efficiency.

Mathematical modelling and statistical analysis assays permitted to establish the optimal conditions for CLF biotransformation with *Trametes pubescens* selected strains, in order to obtain a rate of biodegradation of 60%. From the obtained results it can be concluded that the optimum conditions for CLF degradation were when cultivation took place in aerobic conditions, in minimal liquid medium supplemented whit 3 g·L^−1^ yeast extract, 15 g·L^−1^ peptone, 5 g·L^−1^ glucose, at pH 5.5 inoculated with 2% (*v/v*) vegetative inoculum, after 14 days of cultivation at 25 °C and 135 rpm.

The maximum experimental response for biotransformation rate of CLF was 60% whereas the predicted value was 58.21% indicating a strong agreement between mathematical model and real biotechnological process.

The research approaches would be useful for the practical applications of white-rot fungi in wastewater treatments for xenobiotic recalcitrant compounds removal, as well pharmaceutical pollutants. Moreover, it will be also important to evaluation the adaptation of the selected strains to natural conditions in competition with wild microbiota from the activated sludge.

## 5. Conclusions

This study has scientific and practical importance because offers valuable data regarding to the applying PBD and RSM-CCRD techniques for optimize CLF biodegradation in submerged cultivation using a selected *Trametes pubescens* strain. However, to the best of our knowledge, no report was obtained on the optimization of the culture conditions for CLF biodegradation by white rot fungi belong to species *Trametes pubescens*.

It was demonstrated that nitrogen sources (yeast extract and peptone) concentration, inoculum concentration and incubation time are the significant influence on CLF removal from the liquid medium, in aerobic submerged cultivation conditions. Also, a concentration of 5 g·L^−1^ glucose in cultivation medium composition is effective for CLF biotransformation by *Trametes pubescens* strain. The proposed model illustrates the quantitative effect of variables and the interaction between studied independent variables order to stimulate the metabolic functionality of selected strain for CLF biotransformation during the submerged cultivation in controlled biotechnological conditions. The optimal CLF biodegradation conditions predicted by mathematical modelling were validated by additional experimental results. Thus, a yield of 60% CLF biodegradation is possible to be obtained with *Trametes pubescens* strain, by cultivation in a minimal liquid medium supplemented with glucose (5 g·L^−1^), yeast extract (3 g·L^−1^) and peptone (15 g·L^−1^), inoculated at 2% (*v*/*v*), after 14 days of aerobic submerged cultivation, at 25 °C and 135 rpm.

## Figures and Tables

**Table 1:** The PBD for screening independent variables for CLF biodegradation by *Trametes pubescens*.

Variables	Units	Symbol	Coded Levels (*χ_i_*)	
			−1	+1
Glucose	g·L^−1^	A	5.0	10.0
Yeast extract	g·L^−1^	B	1.0	5.0
Peptone	g·L^−1^	C	10.0	20.0
CuSO_4_·5H_2_O	g·L^−1^	D	0.1	0.5
MnSO_4_·H_2_O	g·L^−1^	E	0.1	0.5
Inoculum level	% *v/v*	F	0.5	2.0
CLF concentration	mg·L^−1^	G	10.0	15.0
Incubation time	days	H	7.0	14.0

**Table 2:** Coded and actual values of independent variables used in the RSM.

Independent Variables	Coded Levels					
	Symbol	−α	−1	0	+1	α
Concentration of yeast extract, g·L^−^^1^	B	0	1	3	5	7
Concentration of peptone, g·L^−^^1^	C	5	10	15	20	25
Inoculum concentration, % (*v/v*)	F	0.2	0.5	1.25	2	2.75
Incubation time, days	H	4	7	10.5	14	17.5

**Table 3:** The PBD matrix for evaluating of biotechnological factors whit influence on CLF biodegradation by *Trametes pubescens* selected strain.

Run	Coded Levels of Variable								Biodegradation Yield of CLF, (%)
	A	B	C	D	E	F	G	H	
1	−1	1	1	1	−1	1	1	−1	30.00
2	−1	−1	−1	−1	−1	−1	−1	−1	40.00
3	−1	−1	−1	1	1	1	−1	1	70.00
4	1	−1	−1	−1	1	1	1	−1	30.00
5	1	1	1	−1	1	1	−1	1	100.00
6	1	1	−1	1	1	−1	1	−1	40.00
7	−1	1	−1	−1	−1	1	1	1	40.00
8	1	−1	1	1	−1	1	−1	−1	40.00
9	1	−1	1	−1	−1	−1	1	1	20.00
10	−1	−1	1	1	1	−1	1	1	25.00
11	−1	1	1	−1	1	−1	−1	−1	40.00
12	1	1	−1	1	−1	−1	−1	−1	75.00

**Table 4:** Experimental design with experimental and predicted values of CLF biodegradation yield by *Trametes pubescens*.

Run	Independent Variable Variation, Coded Levels				CLF Biotransformation Yield, (%)	
	B	C	F	H	Experimental Values	Predicted Values
1	0	0	0	0	47.18	46.67
2	0	0	2	0	60.25	56.67
3	0	0	0	0	47.20	46.67
4	0	0	−2	0	0.00	2.00
5	1	1	−1	1	49.30	43.13
6	−1	1	1	1	59.88	60.63
7	0	2	0	0	36.20	35.83
8	−1	−1	1	−1	21.40	22.29
9	0	0	0	0	47.78	46.67
10	−1	−1	−1	−1	28.25	22.29
11	1	−1	−1	−1	19.97	14.79
12	−1	1	−1	1	29.35	28.54
13	−1	−1	1	1	47.20	46.87
14	0	0	0	0	47.25	46.67
15	1	1	1	−1	30.35	33.13
16	1	−1	1	−1	30.03	29.38
17	0	−2	0	0	25.35	25.38
18	−1	1	−1	1	17.80	16.46
19	2	0	0	0	55.99	56.67
20	1	−1	−1	1	43.99	44.38
21	−2	0	0	0	35.00	35.00
22	0	0	0	2	60.99	61.67
23	1	1	−1	−1	13.20	11.04
24	0	0	0	0	49.25	46.67
25	0	0	0	0	49.45	46.67
26	1	−1	1	1	60.25	53.96
27	−1	1	1	−1	30.25	33.54
28	0	0	0	−2	0.00	2.00
29	1	1	1	1	60.98	59.21
30	−1	−1	−1	−1	15.30	12.71

**Table 5:** Analysis of variance for the response surface of the quadratic polynomial model.

Source	Sum of Squares	Degree of Freedom	Mean Square	*F* Value	*p* Value Prob > *F*
Model	10544.58	14	753.18	15.36	<0.0001
B	704.17	1	704.17	14.36	0.0007
C	150.00	1	150.00	3.06	0.0018
F	3266.67	1	3266.67	66.63	<0.0001
H	4816.67	1	4816.67	98.24	<0.0001
BC	56.25	1	56.25	1.15	0.3010
BF	25.00	1	25.00	0.51	0.0361
BH	400.00	1	400.00	8.16	0.0120
CF	56.25	1	56.25	1.15	0.0310
CH	6.25	1	6.25	0.13	0.7260
FH	225.00	1	225.00	4.59	0.0490
B^2^	1.19	1	1.19	0.024	0.8782
C^2^	429.76	1	429.76	8.77	0.0097
F^2^	304.76	1	304.76	6.22	0.0248
H^2^	304.76	1	304.76	6.22	0.0248
Residual	735.42	15	49.03	–	–
Lack of fit	602.08	10	60.21	2.26	0.1908
Pure error	133.33	5	26.67	–	–
Total	11,280.00	29	–	–	–
